# Multivariate Phenotypic Divergence Due to the Fixation of Beneficial Mutations in Experimentally Evolved Lineages of a Filamentous Fungus

**DOI:** 10.1371/journal.pone.0050305

**Published:** 2012-11-21

**Authors:** Sijmen E. Schoustra, David Punzalan, Rola Dali, Howard D. Rundle, Rees Kassen

**Affiliations:** 1 Department of Biology, University of Ottawa, Ottawa, Ontario, Canada; 2 Laboratory of Genetics, Wageningen University, Wageningen, The Netherlands; 3 Royal Ontario Museum, Toronto, Ontario, Canada; 4 McGill University, Montreal, Quebec, Canada; University of Arkansas, United States of America

## Abstract

The potential for evolutionary change is limited by the availability of genetic variation. Mutations are the ultimate source of new alleles, yet there have been few experimental investigations of the role of novel mutations in multivariate phenotypic evolution. Here, we evaluated the degree of multivariate phenotypic divergence observed in a long-term evolution experiment whereby replicate lineages of the filamentous fungus *Aspergillus nidulans* were derived from a single genotype and allowed to fix novel (beneficial) mutations while maintained at two different population sizes. We asked three fundamental questions regarding phenotypic divergence following approximately 800 generations of adaptation: (1) whether divergence was limited by mutational supply, (2) whether divergence proceeded in relatively many (few) multivariate directions, and (3) to what degree phenotypic divergence scaled with changes in fitness (i.e. adaptation). We found no evidence that mutational supply limited phenotypic divergence. Divergence also occurred in all possible phenotypic directions, implying that pleiotropy was either weak or sufficiently variable among new mutations so as not to constrain the direction of multivariate evolution. The degree of total phenotypic divergence from the common ancestor was positively correlated with the extent of adaptation. These results are discussed in the context of the evolution of complex phenotypes through the input of adaptive mutations.

## Introduction

The study of adaptive evolution has, in recent years, proceeded along two largely independent lines. One approach tracks the evolution of fitness in large populations of asexual microbes in which all new genetic variation is, by design, introduced through naturally arising mutations. The other uses information on phenotypes, which may be correlated with fitness to varying degrees, from natural or laboratory populations of larger, multicellular and generally sexual organisms to make inferences about the strength and form of selection. Although a small number of microbial studies have attempted to connect changes in fitness directly to changes in phenotype (e.g. [1], [2], [3], [4]), and a few phenotype-based studies have examined the role of mutation in generating phenotypic divergence (e.g. [5], [6], [7], [8]), for the most part these two approaches to studying adaptive evolution have proceeded independently. As a first step towards a more unified view of the impacts of adaptive evolution on phenotypes, we present the results of a multivariate phenotypic analysis of the response to selection during replicated experimental evolution of microbial populations.

When considering the evolution of multiple (*n*) traits simultaneously among multiple populations/lineages, population differentiation can be described in a symmetrical *n* × *n* covariance matrix called the divergence (or **D**) matrix. The diagonal elements of **D** contain the variances among population trait means and the off-diagonals represent the covariances among population means for each bivariate trait combination [5], [9], [10], [11], [12]. Insight into patterns of multivariate trait evolution may be gained by characterizing the dominant axes of such covariance matrices via eigenanalysis [13]. This produces a set of orthogonal dimensions of variation, whose directions are described by their eigenvectors and the extent of variation along each are described by the corresponding eigenvalues. The distribution among the latter can be informative regarding the effective dimensionality of the matrix (i.e. its ‘rank’). The rank of **D** in particular describes the extent to which divergence tends to occur in relatively few or many phenotypic dimensions. That is, analyses of rank can reveal whether phenotypic evolution was restricted in its trajectory (i.e. a **D**-matrix of low rank), at the extreme occurring in only a single trait combination (i.e. **d**_max_, the first or leading eigenvector of **D**), or whether it occurred in multiple independent phenotypic directions (i.e. independent trait combinations, reflected in a **D**-matrix of high rank).

Although the analysis of **D**-matrices has been successfully employed in comparative studies of extant populations (e.g. [14], [15], [16]), its application to experimentally evolved populations has been overlooked. To date, most considerations of multivariate divergence have aimed to distinguish the role of selection from genetic constraints by asking whether the principal axis of **D** is biased towards **g**_max_, the axis of greatest genetic variance among these traits [9], [10], [12], [14], [17], [18]. To our knowledge, however, there have been no previous empirical investigations of how the **D**-matrix behaves under varying degrees of mutational input, despite the fact that mutations are recognized as the primary source of novel variation in theoretical treatments of multivariate divergence [5], [8], [19], [20], [21], [22]. The dimensionality of divergence arising from the fixation of beneficial mutations can provide valuable insights into the contribution of mutational covariances to multivariate evolution. For example, trade-offs, arising from strong and consistent antagonistic pleiotropic effects of new mutations on separate traits, could greatly restrict multivariate phenotypic divergence. On the other hand, if the pleiotropic effects of new mutations are generally weak, or highly variable in their strength and sign, phenotypic divergence may proceed essentially unconstrained.

Here we use experimental evolution to quantify phenotypic divergence among replicate populations of a filamentous fungus, *Aspergillus nidulans*, during adaptation to a novel environment. As in other microbial evolution experiments, adaptation proceeds through the substitution of beneficial alleles that arise by mutation and are fixed by selection [23]. Because population size is finite and regularly reduced during transfer to fresh media, mutant alleles with neutral or even deleterious effects can fix through drift or, more likely, hitchhiking. In particular, we used 60 replicate evolved populations, all derived from a single ancestral genotype, that were propagated over approximately 800 generations by periodic transfer to fresh medium under one of two different population size treatments (large or small). This difference in population size was achieved by manipulating the size of the inoculum at each transfer. Such a manipulation may have a number of effects on the dynamics of the evolutionary process, with larger populations having a greater mutational supply, reduced effects of genetic drift relative to selection, and increased clonal competition relative to smaller populations. Previous work has shown that the difference in population size in the current experiment resulted in a significant difference in the extent of adaptation, with final fitness being higher on average in the larger populations [23]. Here, after approximately 800 generations of evolution, we evaluate patterns of phenotypic divergence among these populations in a suite of four characters that capture major features of the *A. nidulans* life-cycle. *A priori*, several of the characters were suspected to be important components of fitness and thus likely targets of selection under the conditions of the experiment [23]. Previous work had also provided evidence of trade-offs between pairs of these traits [24], [25].

We were interested in characterizing patterns of phenotypic divergence with respect to three specific issues. The first concerns the potential effects of population size on the among-population diversification. This was assessed by comparing the mean phenotype and direction of divergence observed in two treatments differing in population size during experimental evolution. The second relates to the dimensionality of adaptation in multivariate trait space. We addressed this by evaluating the rank of the phenotypic divergence matrix **D**. Our final interest was in how phenotypic divergence scales with adaptation, and we addressed this by relating the extent of total phenotypic distance between evolved types and the ancestor to the observed increase in fitness.

## Methods

### Experimental System

We used 60 strains from a recent experiment, first described by Schoustra et al. (2009) [23], in which adaptation to a novel laboratory environment (a rich medium to which the founding genotype initially was maladapted due to a fungicide resistance mutation) occurred due to the fixation of novel beneficial mutations in 112 independently evolving replicate lineages of *Aspergillus nidulans* over approximately 800 generations. Populations were founded from a single ancestral genotype and propagated at two different population sizes by transferring approximately 500 or 50,000 individuals (large and small bottleneck treatment) to fresh medium roughly every 80 generations [23]. During the evolution experiment, lineages adapted to novel conditions and showed variation in terms of the fitness gains achieved (i.e. the degree of adaptation). Analysis of fitness trajectories using a maximum likelihood framework, combined with sexual crosses, demonstrated the fixation of one to three beneficial mutations within each lineage [23]. We expect that at least some (perhaps most) mutant alleles that were fixed are beneficial given the changes in fitness we have observed, but this does not preclude some mutations with neutral, or even deleterious, effects fixing through drift or, more likely, hitchhiking. Fitness was measured as mycelial growth after 5 days (MGR), a common measure of absolute fitness in filamentous fungi [26], [27], [28] and which is strongly correlated with the outcome of competitive fitness assays in these genotypes [23]. To evaluate the phenotypic divergence that accompanied adaptation in these lineages, we assayed a subset of each population size treatment (30 lineages each from the small and large bottleneck), as well as the ancestral genotype, after 800 generations. For the large population size treatment we had 7 strains that had fixed one beneficial mutation, 12 with two beneficial mutations and 11 with three beneficial mutations. For the small population size treatment, we had 12 strains with one beneficial mutation, 11 with two beneficial mutations and 7 with three beneficial mutations.

Based on the life-cycle of *A. nidulans* (see Text S1), we chose to measure the following phenotypic characters of three independent replicates (i.e. colonies) for each trait in each lineage: (1) biomass – BM – as the total biomass a fungal colony produces per surface area, including mycelium, sporeheads, sexual fruiting bodies and spores; (2) density of nuclei from the mycelium measured as colony forming units – CFU, (3) fraction fast germinating spores, indicative of the percentage diploids – DPL –and providing a measure of the equilibrium within the parasexual cycle between haploid and diploid spores (usually 99.9% haploid); and (4) sexual fruiting bodies – SFB – as a measure how often the sexual cycle is completed. We also measured fitness (MGR) in triplicate for all genotypes used in this study. The latter is a repetition of the assay presented previously in [23]. Details on how the actual measurements were performed are provided in Text S2.

### Measuring the Geometry of Phenotypic Divergence

In a few cases extreme phenotypes were observed that may represent statistical outliers. However, re-analysis with such data points excluded (not shown) had no qualitative effect on the results or significance tests, so we report analyses based on the complete dataset. Traits were individually standardized (mean = 0, standard deviation = 1) across lineages prior to analyses. We implemented a multivariate mixed model, fit via Restricted Maximum Likelihood, using the MIXED procedure in SAS v. 9.2 (SAS Institute, Cary NC). Variation in the four phenotypic traits was modeled as:
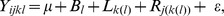
where *Y* is the observed value of trait *i* from the *j*th replicate (*R)* nested within the *k*th lineage (*L)* nested within the *l*th population size treatment (*B)*. Replicate, lineage, and the residual error (*ε*) are random effects; fixed effects include the intercept (*μ*) and population size treatment (*B_l_*).

An estimate of the pooled (i.e. across population size treatments) divergence matrix, **D**, is provided by the lineage-level covariance matrix. To determine the dimensionality of **D**, we used a factor-analytic modeling approach in which **D** was constrained to be from four to zero dimensions and a series of nested likelihood ratio tests were used to determine the significance of including/excluding dimensions [29], [30].

The population size effect (*B*) tests for a difference between treatments in average multivariate phenotype. To test for treatment differences in the covariance structure of **D**, we used a likelihood ratio test to compare the fit of the above model (i.e. estimating a single, pooled **D**) to one that estimated separate covariance matrices at the lineage level by employing the group statement in PROC MIXED. An unconstrained covariance matrix (i.e. “type = un” in the ‘repeated’ statement) was fit at the lineage level in all cases in this analysis. A similar analysis (not shown) that considered the effect of the number of mutations fixed (i.e. 1, 2, or 3, as estimated in [23]) provided no evidence of significant variation in the covariance structure of **D** among these groups.

As a complementary approach to evaluating treatment differences in **D**-matrix structure, we used CPCA (common principal component analysis; [31], [32]) to compare treatment-specific **D**-matrices calculated from the (unstandardized) lineage-specific mean trait values. This allowed testing of several hypotheses of matrix structure that includes more subtle forms of similarity, including matrix equality, proportionality and common eigenstructure. We used a model building approach with the Akaike’s Information Criterion to assess the best model of matrix similarity [32]. CPCA analyses were conducted using software provided by P. Phillips (http://pages.uoregon.edu/pphil/programs/cpc/cpc.htm).

In recognition of growing concerns in the literature surrounding appropriate data standardization, we repeated these matrix comparisons using mean-standardized estimates of (co)variance which are sometimes more appropriate when trait means/variances differ considerably in scale [33], [34]. Since our results were qualitatively consistent regardless of which approach was used, all reported analyses are based on the variance-standardized values. Trait means (pooled across treatments and including the ancestral phenotype) are provided in Table 1 to facilitate calculation of mean-standardized values.

**Table 1:** Pooled (across treatments) divergence (**D**)-matrix calculated from trait means, prior to variance-standardization, for 60 experimentally evolved lineages.

Trait					
	BM	CFU	DPL	SFB	Mean
**BM**	1.727	**0.464**	**0.180**	**−0.001**	7.46
**CFU**	0.464	0.881	**0.257**	**−0.035**	21.71
**DPL**	0.291	0.297	1.518	**−0.367**	3.88
**SFB**	**−**0.002	**−**0.049	**−**0.682	2.271	3.87

Variances (diagonal), covariances (below) and correlations (above) are based on the lineage means for four traits: biomass (BM), density of nuclei (CFU), percent diploids (DPL) and sexual fruiting bodies (SFB). Except for BM, trait values were ln-transformed prior to analysis. Trait mean values also include the ancestral phenotype (n = 61).

### Relating Phenotypic Divergence to Fitness

To confirm that the measure of fitness (MGR) of a particular genotype obtained in the present study were comparable to those obtained previously [23], we calculated the Pearson’s correlation (*r*) between mean fitness measures for 56 lineages (data were unavailable for four lineages appearing in [23]).

To test the degree to which multivariate phenotypic divergence reflected adaptive evolution, we first recalculated **D** when including trait values of the common ancestor, and then subsequently calculated the Mahalanobis distance (**d_i_**) between each evolved lineage and the ancestral phenotype as:
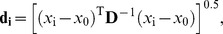
where *x*_i_ represents the column vector of mean trait values for the *i*th evolved lineage, *x_0_* is the vector of mean trait values for the ancestral genotype (averaged across the three replicate ancestral isolates) and **^T^** indicates matrix transposition. Mahalanobis distance appropriately scales among-lineage differences according to the variability of each trait as well as the covariances between traits [35]. Thus, **d_i_** represents a unit-free measure of total phenotypic divergence from the common ancestor. Subsequently, we evaluated the correlation between (ln-transformed) mean phenotypic distance (**d_i_**) and adaptation (i.e. mean fitness of the evolved lineage relative to the ancestor in each evolved line, following [23]). Estimating the relationship between phenotypic distance and adaptation directly from the raw values (i.e. instead of lineage means), while accounting for the population size and lineage effects, provided qualitatively identical results (not shown). We looked for evidence of an interaction between population size treatment and distance using an ANCOVA with population size and **d_i_** as predictor variables (including their interaction) and adaptation (fitness) as the response variable. To visually contrast the treatments with respect to the relationship between adaptation and multivariate divergence, we plotted the mean fitness of each lineage against its scores on the first two principal component axes describing the combined multivariate phenotypic space (i.e. Principal Components Analysis of the pooled divergence matrix, but including the ancestral mean phenotype; n = 61). These two axes summarized approximately 74% (i.e. 43% and 31%) of the total phenotypic variation.

Statistical analyses and mathematical operations were performed using JMP v. 5.0.1a (SAS Institute, NC), the Poptools add-in for Excel (available at http://www.cse.csiro.au/poptools), and the base package in R: A Language and Environment for Statistical Computing (available at: http://www.R-project.org).

## Results

### Effect of Population Size on Phenotypic Divergence

**Figure 1:**
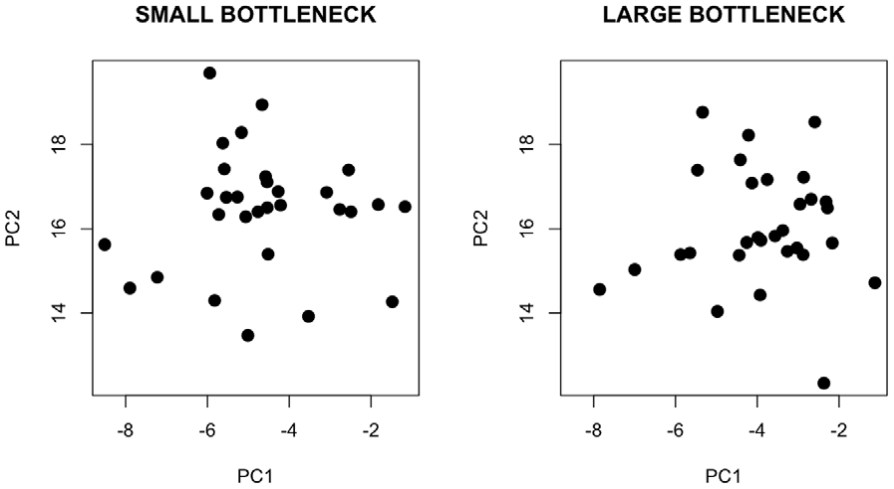
Observed dispersion of phenotypic means along the first two canonical axes (PC1 and PC2) of the pooled divergence matrix for small (left panel) and large (right panel) population size treatments. These two axes summarized approximately 74% (i.e. 43% and 31%) of the total phenotypic variation.

Figure 1 shows the observed dispersion of phenotypic trait means along the first two canonical axes of the pooled divergence matrix **D** for both population size treatments. We detected significant among-population (i.e. lineage) divergence in phenotypic trait means, evident as a significant proportion of variation explained by the first dimension of the pooled **D**-matrix (i.e. the minimum amount of genetically-based divergence; Tables 1, 2, 3). However, trait means did not differ consistently between the two population size treatments (population size effect: *F*_1,234_ = 1.32, p = 0.251) and we found no evidence for differences in their respective **D**-matrices (likelihood ratio test: χ^2^ = 9.00, df = 10, p = 0.532). Consistent with these results, the CPCA model-building approach indicated matrix equality as a better fit than proportionality (Table 4). Thus, the two population size treatments did not significantly differ in any aspect of their multivariate divergence and we proceeded to characterize the overall pattern (i.e. dimensionality) of divergence from a single, combined **D**-matrix (Table 3).

**Table 2:** Summary of the eigenanalysis of the **D**-matrix based on trait means for all evolved lineages (n = 60).

	λ	proportion	BM	CFU	DPL	SFB
V1	**2.743**	0.43	**−**0.231	**−**0.164	**−**0.537	0.795
V2	**1.984**	0.31	0.822	0.367	0.144	0.411
V3	**1.034**	0.16	**−**0.415	0.128	0.787	0.438
V4	**0.637**	0.10	**−**0.315	0.907	**−**0.267	**−**0.085

For each of the respective eigenvectors (V1–V4), the table shows corresponding eigenvalues (**λ**), the proportion of total divergence each eigenvector explains and trait loadings for each of the traits.

**Table 3:** Results of nested likelihood ratio tests assessing the effective dimensionality of the pooled **D**-matrix.

Number of dimensions	−2 log likelihood	Number of parameters	AIC	P
4	1670.3	20	1687.0	0.0008
3	1681.45	19	1719.5	<0.0001
2	1726.5	17	1758.5	<0.0001
1	1786.5	14	1812.5	<0.0001
0	1925.8	10	1945.8	–

The table shows P-values for log-likelihood ratio tests, indicating whether adding an dimension significantly improves the fit of the model with given number of assumed dimensions (k) to the model directly below (k-1 dimensions). AIC indicates Akaike’s Information Criterion.

**Table 4:** Results of Flury decomposition (CPCA) for tests of matrix similarity between divergence matrices derived from small and large population size treatments.

Model comparison						
higher	lower	χ^2^	DF	P	χ^2^/df	AIC
Equality	Proportionality	1.818	1	0.1775	1.818	7.653
Proportionality	CPC	1.343	3	0.7191	0.448	7.835
CPC	2 CPCs	1.688	1	0.1939	1.688	12.493
2 CPCs	1 CPCs	2.218	2	0.3299	1.109	12.805
1 CPCs	unrelated	0.587	3	0.8994	0.196	14.587
unrelated						20.000

### Number of Phenotypic Dimensions

We found statistical support for a **D**-matrix of full rank (Tables 2 and 3), indicating substantial and statistically significant divergence in all four phenotypic dimensions.

### Relationship between Adaptation and Phenotypic Divergence

**Figure 2:**
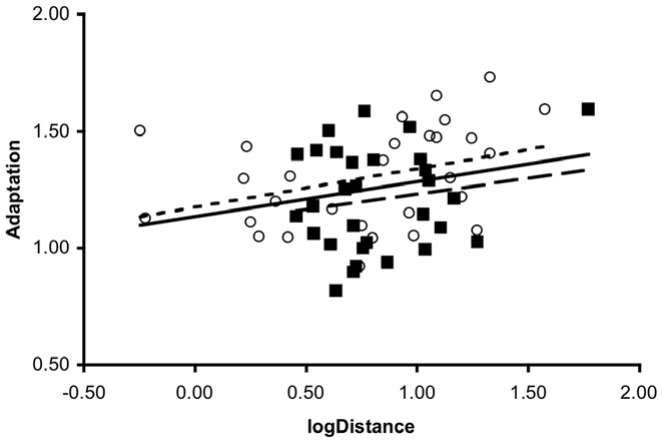
Bivariate plot of observed (log_e_) mean phenotypic distance versus adaptation for evolved lineages. Closed squares indicate data for the large population size treatment, open circles indicate those for the small population size treatment. The solid line indicates the major-axis regression for both treatments, pooled. The dashed lines give the major axis regressions for the two population size treatments separately; the small dashes for the small population size treatment, the large dashes for the large population size treatment.

The degree of overall phenotypic divergence (**d_i_**) observed in each evolved strain was significantly correlated with the extent of adaptation (Figure 2; *r* = 0.262, p = 0.043, n = 60; See Figure S1 for 3D plots). Though we found no significant treatment differences in the slope of the relationship between adaptation and phenotypic distance (treatment × distance interaction: *F*_1,1_ = 0.321, p = 0.573), this relationship was pronounced in the small population size treatment (*r = *0.358, p = 0.052, n = 30) but less so in the large population size treatment (*r* = 0.174, p = 0.359, n = 30). Although one lineage in the large population size treatment showed a relatively high value of both adaptation and phenotypic distance (see Fig. 2), reanalysis after omitting this data point did not qualitatively change the interpretation (i.e. no significant relationship between adaptation and distance in the large population size treatment, and no significant treatment × slope interaction). Further examination, using a linear mixed model, with adaptation as a response variable and with population size treatment and the four traits as predictors (Table S1), suggested that the relationship between phenotypic distance and adaptation was largely the result of a relationship between adaptation (measured as MGR) and CFU, suggesting the latter as a direct target of selection (i.e. reduction of CFU in relation to an increased MGR; [25]).

## Discussion

We have evaluated the extent to which initially identical replicate lineages allowed to adapt through the fixation of (beneficial) mutations also diverged in multivariate phenotype space. Our main results are: (1) Reducing the population size, which should result in a reduced mutation supply, has no effect on the overall pattern of phenotypic divergence; (2) adaptive phenotypic divergence occurred in all four measured directions; (3) the amount of phenotypic divergence scaled with adaptation in terms of fitness increase relative to the ancestral genotype. We discuss each of these results in turn below.

### Effect of Population Size on Phenotypic Divergence

Our observation that phenotypic divergence was unaffected by the population size, both in the average divergence among multivariate phenotypes and in the principal directions of phenotypic variance, was unexpected. The simplest explanation is that effective population sizes were far more similar between treatments than the census sizes imposed during transfer. Such an interpretation is inconsistent, however, with our previously demonstrated effects of the population size treatments on final fitness [23].

Theory suggests that the effects of drift should be exaggerated when population sizes are small, so we might have expected greater divergence in multivariate phenotype among the small as compared to the large populations. Although such a trend was observed (i.e. variation in **d**_i_ is greater among small than large populations; Fig. 2), it was insufficient to generate a significant difference in **D**. This may simply represent insufficient power to detect significant differences in second-order effects (i.e. variances and covariances). Alternatively, while fitness may respond in predictable ways to changes in population size, the pleiotropic effects of the beneficial mutations on our measured phenotypes may have been sufficiently variable that phenotypic divergence was unaffected.

### Dimensionality of Divergence

We observed a pattern of divergence with maximal dimensionality, or in other words, the independent lineages evolved in all directions in phenotype space. Divergence was also not strongly biased in any particular direction, as can be seen from the distribution of eigenvalues and the corresponding proportion of variance explained by each (Table 2). Since each of the four characters we measured was chosen to capture a different aspect of the *A. nidulans* life-cycle, this suggests that selection acted on mutations that affect all of these. These results also suggest that increases in fitness can be achieved through a wide range of distinct phenotypic routes and importantly, that pleiotropy did not impose a major constraint on phenotypic divergence. This abundance of available phenotypic solutions indicates that mutational covariances were either weak overall, or sufficiently variable such that systematic trade-offs did not constrain the response to selection. The latter may arise from the diverse ways a particular mutation may interact with the background genetic and developmental system [36], [37], [38], [39], [40], [41], [42], [43]. Our results further suggest that genetic architecture underlying covarying phenotypes is likely to be evolutionarily labile [44].

That increases in fitness can be achieved through a range of distinct phenotypic routes is consistent with what we have previously seen when examining among-lineage variation in final fitness [23]. It is also consistent with the existence of a rugged underlying adaptive landscape. Taken on their own, however, these phenotypic results do not exclude the possibility of a single smooth and broad landscape that lacks any distinct peaks or valleys.

### Relationship between Adaptation and Phenotypic Divergence

We found a positive correlation between the degree of multivariate phenotypic divergence (i.e. distance) and the extent of adaptation (i.e. mean fitness) across lineages. This result is perhaps not surprising, since we chose traits that were suggested to be important correlates of fitness. Further examination indicated that much of the observed relationship between adaptation and phenotypic divergence was driven by a negative relationship between fitness and colony forming units (CFU), and fitness and the density of sexual fruiting bodies (SFB) (also see [24], [25]). This could be a consequence of experimental conditions that selectively favoured a reduction in the density of nuclei or, alternatively, indicative of allocation to other components of fitness at the expense of CFU. The high dimensionality of divergence is consistent with many phenotypic combinations (resulting from different mutations) that achieve this adaptive reduction.

Interestingly, we found some evidence that the relationship between adaptation and divergence was more prominent in the small population size treatment than in the large population size treatment. Multivariate phenotypic distance from the ancestor was also more variable among lineages in the small than the large population size treatment (Fig. 2), a pattern that is also reflected in the traces of the separate **D** matrices but was insufficient to generate a significant difference in them.

At least three explanations for this effect seem plausible. One is that the spectrum of mutations available to selection differs among population size treatments due to the biasing effects of drift and/or clonal competition. This may be reflected to some extent in the pleiotropic effects these mutations have on the phenotypic traits we have measured. Under this view, the difference in the relationship between adaptation and phenotypic divergence we observe here is an idiosyncratic effect of the mutations that are substituted. A second explanation is that the effects of drift and hitchhiking may be more pronounced at small population sizes since selection will in effect be weaker, allowing a larger fraction of neutral or mildly deleterious alleles to escape elimination. A third explanation is that populations that spend longer periods of time at an adaptive peak, as might be the case with lineages from the large population size treatment, have more opportunity to accumulate mutations with neutral fitness effects. Since the total neutral phenotypic divergence is proportional to mutational (co)variance and time [5], [8], [20], see [45], eq. 7, we would expect the covariance between phenotypic distance and adaptation to degrade over time as a consequence of neutral processes. Final fitness was higher on average in the larger populations [23], consistent with the idea that the small populations may be further from an adaptive peak. However this does not seem to be an appropriate explanation for our results as the vast majority of lines had reached a fitness plateau by the end of the experiment [23].

### Summary

Studies on the dynamics of adaptation have frequently adopted the ‘adaptive landscape’ (sensu Wright [46] and Simpson [47]) metaphor often used in theoretical models of adaptation, whereby a fitness optimum is a function of phenotypic values [19], [21], [48], [49], [50]. Collectively, our results suggest an adaptive landscape with many available evolutionary paths, access to which is not constrained by pleiotropic effects of the beneficial mutations fixed. Overall, our study highlights the utility of employing multivariate analysis of phenotypic divergence and the importance of relating observed divergence to adaptation. Moreover, we demonstrate the value of integrating approaches used by subfields of evolutionary biology that, previously, have been somewhat disconnected.

## Supporting Information

Figure S1 **Three-dimensional plots of phenotypic divergence of lineages evolved under small (open circles) and large (closed squares) bottleneck treatments with respect to the ancestral phenotype (marked as an “x” and placed at the origin, denoted by a dashed line).** Trait distances (in units of standard deviations) are for biomass (BM), colony forming units (CFU), percent diploids (DPL) and sexual fruiting bodies (SFB).
(JPG)

Table S1 **Parameter estimates and corresponding effects from linear mixed model regression of adaptation on population size treatment and traits.**
(DOC)

Text S1 **Model system.**
(DOC)

Text S2 **Targets of selection during experimental evolution.**
(DOC)
