# Supplementary figures and images for: Multivariate Phenotypic Divergence Due to the Fixation of Beneficial Mutations in Experimentally Evolved Lineages of a Filamentous Fungus
Source: PLoS One. 2012 Nov 21;7(11):e50305. doi: 10.1371/journal.pone.0050305 (PMC3504003; doi:10.1371/journal.pone.0050305)

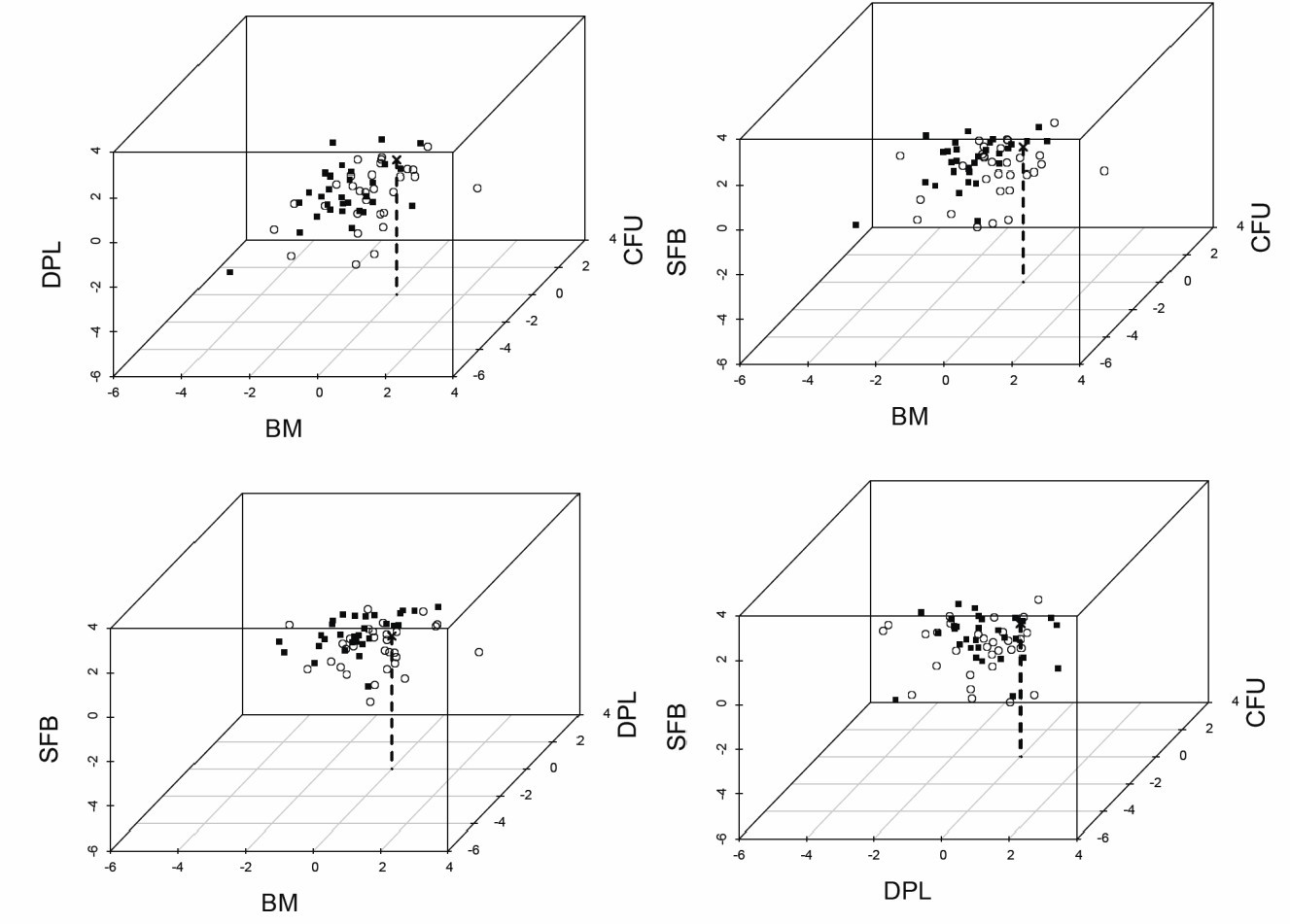

Supplement: Figure S1 — Three-dimensional plots of phenotypic divergence of lineages evolved under small (open circles) and large (closed squares) bottleneck treatments with respect to the ancestral phenotype (marked as an “x” and placed at the origin, denoted by a dashed line). Trait distances (in units of standard deviations) are for biomass (BM), colony forming units (CFU), percent diploids (DPL) and sexual fruiting bodies (SFB). (JPG) [file pone.0050305.s001.jpg]
