# Supplementary material for: Multivariate Phenotypic Divergence Due to the Fixation of Beneficial Mutations in Experimentally Evolved Lineages of a Filamentous Fungus
Source: PLoS One. 2012 Nov 21;7(11):e50305. doi: 10.1371/journal.pone.0050305 (PMC3504003; doi:10.1371/journal.pone.0050305)
Supplement: Table S1 — Parameter estimates and corresponding effects from linear mixed model regression of adaptation on population size treatment and traits. (DOC) [file pone.0050305.s002.doc]

**Table S1.** Parameter estimates and corresponding effects from linear mixed model regression of adaptation on population size treatment and traits. SE = Standard error of estimate, df = degrees of freedom, SS = Sums of Squares.

|  | Estimate | SE | t-ratio | df | SS | *F* | *p* |
| --- | --- | --- | --- | --- | --- | --- | --- |
|  |  |  |  |  |  |  |  |
| Intercept | 2.776 | 0.641 | 4.33 | -- | -- | -- | -- |
| Population size | -0.043 | 0.032 | -1.33 | 1 | 0.0748 | 1.76 | 0.190 |
| BM | 0.019 | 0.026 | 0.74 | 1 | 0.0230 | 0.54 | 0.465 |
| CFU | -0.079 | 0.032 | -2.47 | 1 | 0.2592 | 6.11 | 0.017 |
| DPL | 0.020 | 0.027 | 0.75 | 1 | 0.0238 | 0.56 | 0.457 |
| SFB | -0.010 | 0.023 | -0.45 | 1 | 0.0084 | 0.20 | 0.657 |
| Population size*BM | 0.011 | 0.026 | 0.44 | 1 | 0.0083 | 0.20 | 0.660 |
| Population size*CFU | 0.044 | 0.032 | 1.37 | 1 | 0.0794 | 1.87 | 0.177 |
| Population size*DPL | 0.007 | 0.027 | 0.28 | 1 | 0.0033 | 0.08 | 0.782 |
| Population size*SFB | 0.030 | 0.023 | 1.35 | 1 | 0.0771 | 1.82 | 0.183 |
