# Supplementary material for: Multivariate Phenotypic Divergence Due to the Fixation of Beneficial Mutations in Experimentally Evolved Lineages of a Filamentous Fungus
Source: PLoS One. 2012 Nov 21;7(11):e50305. doi: 10.1371/journal.pone.0050305 (PMC3504003; doi:10.1371/journal.pone.0050305)
Supplement: Text S1 — Model system. (DOC) [file pone.0050305.s003.doc]

**S1. Model system**

***Aspergillus nidulans* life-cycle.** The traits were selected based on the life cyle of *A nidulans* (see ). Briefly, A fungal colony can start with either a chunk of mycelium containing at least one viable nucleus, an asexually derived conidiospore or a sexually derived ascospore. After several nuclear divisions, hyphal tubes will start developing, giving rise to a structured mycelium where semi-permeable septa separate mycelial apical and subapical cells. Some subapicial cells will specialize to form either a foot cell which will carry a stalk holding a condiophore with conidiospores. Alternatively, subapical cells can initiate a sexual cycle. In the sexual cycle, 2 neighboring nuclei fuse to a diploid that after several mitoses goes through meiosis. Nuclei in the mycelium may also fuse to give rise to a diploid nucleus that can grow vegetatively and form a diploid mycelium carrying diploid conidiospores. Diploids can revert to haploidy by repeated loss of chromosomes in a process called the parasexual cycle.

**Measuring phenotypes and fitness.** This is a repetition of information described in and .

*Preparation of spore suspensions.* 30 µl of a dense spore suspension from stored (-80 ^o^C) evolved lineages of a previous study was spread out on a Petri dish with CM in three-fold. After one week of incubation, every entire plate was scraped off using 5 ml saline tween (water containing NaCl 0.8% and Tween-80 0.05%), yielding a suspension containing mycelium and spores which was vigorously vortexed for 45s. These spore suspensions were used in the subsequent assays.

*Biomass (BM).* Filter papers (Whatman 1001-042; 42.5mm) were weighed on a fine scale before applying 1 ml of spore suspension. After 24h of drying, the filter paper was weighed again, the difference in weight is attributable to the biomass in the suspension.

*Colony forming units (CFU).* Serial dilutions were made of the spore suspensions in a minimal salts solution (Na_2_HPO_4_ 6.7 g, KH_2_PO_4_ 3 g, NaCl 0.5 g, NH_4_Cl 1.0 g, 1000 ml dH_2_O) that were plated on CM supplemented with triton (40 µl/l). Triton reduces colony size of fungal colonies to facilitate counting. After 48h of incubation, colonies were counted and the total number of colony forming units (CFU) in the original suspension was calculated.

*Fraction fast germinating spores including diploids (DPL)*. We estimated the number of diploid nuclei present in the spore suspension using the so-called sandwich method (modified from ). A ten-fold dilution of the suspension was mixed in 3 ml Minimal Medium (set at pH 5.8, consisting of NaNO_3_ 6.0 g/l; KH_2_PO_4_ 1.5 g/l; MgSO_4_.7H_2_O 0.5 g/l; NaCl 0.5 g/l; 0.1 ml of a saturated trace element solution containing FeSO_4_, ZnSO_4_, MnCl_2_, and CuSO_4_; agar 10 g/l and (added after autoclaving) glucose 4.0 g/l and pyridoxine 0.1 mg/l) and poured out in an empty Petri dish. After solidification, an additional sandwich layer of 30 ml Minimal Medium was poured on top. After 24h of incubation, colonies were counted. Since diploids grow substantially faster through the sandwich layer and have another appearance than haploids, diploids can in this way be distinguished from haploids. Using the cfu counts done on the same suspension as described above, the fraction diploids in the suspension was calculated.

*Density of sexual fruiting bodies (SFB).* 30 µl of spore suspension was spread out on a Petri dish with CM in three-fold. After 12 days of incubation – this is when sexual fruiting bodies have fully matured – a picture of each Petri dish was taken using a macro-scope. From the pictures, we counted the number of sexual fruiting bodies in an area of 6.4 x 4.2 mm.

*Fitness.* We measured mycelial growth rate (MGR) in triplicate by placing 5 µl of spore suspension in the centre of a Petri Dish with CM (in the same way as in ). After 5 days of incubation, the diameter of the resulting fungal colony was measured in two peripendicular directions. For each evolved lineage, the extent of adaptation was calculated as the difference between the fitness of each line and the fitness of the ancestor. MGR of all strains was measured in one single assay. Previous work has shown that MGR strongly correlates (positively) with other possible fitness measures such as competitive fitness and production of nuclei over time (). Estimates of fitness were repeatable between studies; mean fitness estimates obtained in the present study were significantly correlated with the estimates obtained for the same subset of lineages used in the previous study by Schoustra et al. (*r* = 0.817, p = 0.009, n = 56). For the subset of lineages used in the present study, the mean fitness was marginally higher in the small population size treatment (t-test: *t*_58_ = 1.747, p = 0.086), apparently an artifact of sampling.

For the subsequent analyses, CFU, DPL and SFB were log_e_ transformed; BM did not require transformation. Examination of pairwise scatterplots revealed some genotypes that could represent bivariate statistical outliers but because of the nature of our study (i.e. estimating phenotypic evolution due to novel beneficial mutations) we felt that these values were biologically meaningful and did not remove them from subsequent analyses.
