# Supplementary material for: Multivariate Phenotypic Divergence Due to the Fixation of Beneficial Mutations in Experimentally Evolved Lineages of a Filamentous Fungus
Source: PLoS One. 2012 Nov 21;7(11):e50305. doi: 10.1371/journal.pone.0050305 (PMC3504003; doi:10.1371/journal.pone.0050305)
Supplement: Text S2 — Targets of selection during experimental evolution. (DOC) [file pone.0050305.s004.doc]

**S2. Targets of selection during experimental evolution.**

To identify possible targets of direct selection during adaptation, we used a linear mixed model with adaptation as a response variable and with population size treatment, the four traits and the treatment by trait interactions as predictors. We found evidence of a negative relationship between adaptation and CFU (Table S1), with no significant treatment x trait interactions. This implicates reduction of CFU as a possible target of selection.
